# Automated Cell Treatment for Competence and Transformation of *Escherichia coli* in a High-Throughput Quasi-Turbidostat Using Microtiter Plates

**DOI:** 10.3390/microorganisms6030060

**Published:** 2018-06-25

**Authors:** Sebastian Hans, Matthias Gimpel, Florian Glauche, Peter Neubauer, Mariano Nicolas Cruz-Bournazou

**Affiliations:** Chair of Bioprocess Engineering, Institute of Biotechnology, Technische Universität Berlin, Ackerstraße 76, D-13357 Berlin, Germany; sebastian.hans@tu-berlin.de (S.H.); matthias.gimpel@tu-berlin.de (M.G.); florian.glauche@tu-berlin.de (F.G.); peter.neubauer@tu-berlin.de (P.N.)

**Keywords:** competent cells, *Escherichia coli*, turbidostat, automation, high throughput, chemostat, transformation

## Abstract

Metabolic engineering and genome editing strategies often lead to large strain libraries of a bacterial host. Nevertheless, the generation of competent cells is the basis for transformation and subsequent screening of these strains. While preparation of competent cells is a standard procedure in flask cultivations, parallelization becomes a challenging task when working with larger libraries and liquid handling stations as transformation efficiency depends on a distinct physiological state of the cells. We present a robust method for the preparation of competent cells and their transformation. The strength of the method is that all cells on the plate can be maintained at a high growth rate until all cultures have reached a defined cell density regardless of growth rate and lag phase variabilities. This allows sufficient transformation in automated high throughput facilities and solves important scheduling issues in wet-lab library screenings. We address the problem of different growth rates, lag phases, and initial cell densities inspired by the characteristics of continuous cultures. The method functions on a fully automated liquid handling platform including all steps from the inoculation of the liquid cultures to plating and incubation on agar plates. The key advantage of the developed method is that it enables cell harvest in 96 well plates at a predefined time by keeping fast growing cells in the exponential phase as in turbidostat cultivations. This is done by a periodic monitoring of cell growth and a controlled dilution specific for each well. With the described methodology, we were able to transform different strains in parallel. The transformants produced can be picked and used in further automated screening experiments. This method offers the possibility to transform any combination of strain- and plasmid library in an automated high-throughput system, overcoming an important bottleneck in the high-throughput screening and the overall chain of bioprocess development.

## 1. Introduction

The vast number of factors that influence the expression of recombinant protein production in bioprocesses makes screening a challenging task in bioprocess development [1]. The choice of the strain is typically made at early stages in product development and is therefore excluded in the following steps [2,3].

With the increasing number of tools to manipulate DNA, new options are available in the field of metabolic engineering, and genome editing [4,5]. On that node, the expression host gets more in focus of the optimization processes [6]. Metabolic engineering to increase the production of small molecules is a common task for various hosts [7,8,9,10]. The availability of a variety of expression plasmids with low (e.g., pSC101 [11]), medium (pBR322 [12]) or high (e.g., pUC18/19 [13]) copy numbers as well as different constitutive and inducible promoter systems (e.g., P_T7_ [14], P_lac_, [15], P_BAD_ [16], P_m_/Xyls System [17]), controlling target gene expression, enlarge the search region for the optimal bioprocess even further.

Beyond the field of bioprocess development, studying knockout mutants helps to get a deeper understanding of gene functions and regulatory processes. With the use of fluorescent reporter systems, genetic networks can be studied. The largest available set of *Escherichia coli* (*E. coli*) strains with unknown behavior is the Keio Collection [18,19]. The Keio Collection is a library of 3864 *E. coli* K-12 single knockout strains. Similar collections are also described for *Bacillus subtilis* [20], *Pseudomonas aeruginosa* [21], *Acinetobacter baylyi* [22] and *Saccharomyces cerevisiae* [23]. With the use of fluorescent reporter systems, genetic networks can be studied in these strain libraries in vitro online and without extensive analytics [24]. Nevertheless, a systematic study of these collections with a reporter system is very difficult without automated treatment. Hence, there is a need for automated and high throughput treatment for cell competence and transformation.

**Figure 1:**
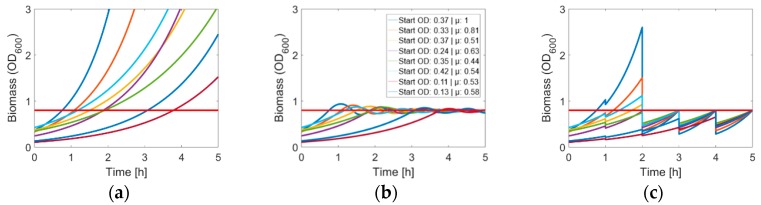
Illustrated overview of possible cultivation modes for the preparation of competent cells. Values for initial biomass and µ were chosen randomly; red line: threshold for harvesting (Optical Density (OD) = 0.8). The used models could be seen in the appendix. (**a**) Batch cultivation; (**b**) chemostat cultivation; (**c**) quasi-turbidostat cultivation.

The easy handling and the well-established molecular and microbiological methods made *E. coli* into one of the most commonly used organisms for heterologous protein production. Until the end of 2011, over 200 biopharmaceuticals have gained regulatory approval, nearly one third of them are produced in *E. coli* [2], demonstrating its importance for biotechnology. The first step in the process of manipulating cells is their treatment for competence. In *E. coli* there are mainly two different methods for competent cell preparation available: chemical treatment with CaCl_2_ [25,26,27,28] or the use of electricity [29,30]. As the competence depends on the physiological state of the cell, for both methods, the cultures must be harvested at a certain turbidity (optical density, OD) during the exponential growth phase. What is a basic task in laboratory with a well-known strain, could be a challenging task in a high throughput screening with a vast number of strains of unknown growth behavior. Whereas the problem of automated competence treatment has been solved when using a single strain [31,32], completely different problems arise when using entire strain libraries. Normally, a batch culture is chosen to start the treatment of cells for competence. The cells are more or less monitored until a certain OD is reached. Even though automated frameworks exist to harvest the culture at a desired biomass concentration [33], different growth rates, starting ODs and lag-phases make it difficult to reach the same OD at the same time when transforming different clones (Figure 1a). From the perspective of bioprocess engineering, the method of choice to maintain the cells at a given condition would be a continuous cultivation [34]. The most used system (due to its simplicity and robustness) is the chemostat, where the growth rate is determined by the dilution rate. An extension of the chemostat is the turbidostat method. Here, the OD is continuously monitored and the dilution is controlled by the OD signal. Such a system enables cultivation close to the maximum growth rate at a specified OD (Figure 1b). However, the experimental setup for such a system is complex, consumes relatively high amounts of media, is prompt to faults in pumps or sensors, and its miniaturization and parallelization is challenging [35]. Even though miniaturized turbidostats have been realized [36,37] the experimental setup is still laborious and the parallelization does not reach the throughput of a 96-microwell plate.

To ensure a constant quality of DNA transformation, we developed a new strategy for optimal preparation of competent *E. coli* cells based on a CaCl_2_ treatment. Here, optimal means that all cells are in the exponential growth phase, the OD is equal, and the desired conditions are maintained up to the selected harvesting time. This new method is an automated, high throughput quasi-turbidostat, developed for 96 well plates (Figure 1c). Furthermore, as proof of concept, we compare the results obtained from manual and automated transformation of different *E. coli* strains.

## 2. Materials and Methods

### 2.1. Experimental Platform

**Figure 2:**
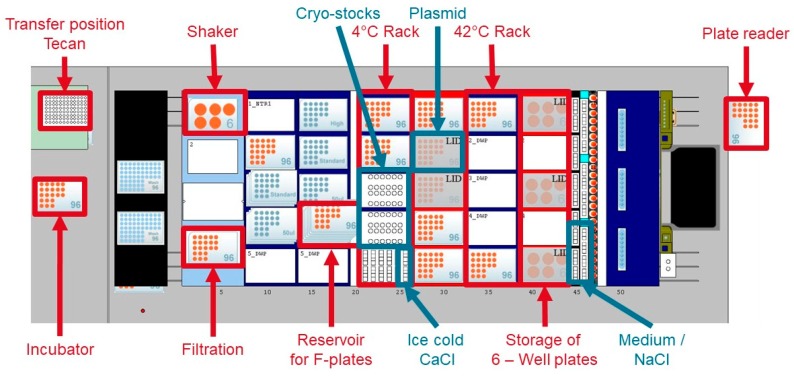
Deck layout of the used Hamilton Microlab Star liquid handling station. In this platform a FAME incubator (Hamilton), a Shaker (inheco Industrial Heating and Cooling GmbH, Planegg, Germany), a vacuum station, two terminable racks (each for five SBS labware) and a Synergy MX II plate reader (BioTek, Bad Friedrichshall, Germany) are mounted. Red: used Labware/Hardware; Blue: provided liquid solutions.

As experimental platform a Hamilton Mircolab Star (Hamilton Bonaduz AG, Bonaduz, Switzerland) is used as described in [38]. Figure 2 gives an overview of the deck layout; the method is archived in the Supplementary Materials (Source Code S1/ Source Code S2). A freedom EVO 200 liquid handling platform from Tecan (Tecan, Männedorf, Switzerland, see Figure S3) is placed back-to-back with the Hamilton platform. Both liquid handlers are connected by a linear transfer unit, controlled by the Hamilton Venus ONE software.

All cultivations are carried out in U-shaped microtiter plates (Greiner Bio-One, Frickenhausen, Germany), incubated at 37 °C and aerated by shaking at 1000 rpm at an amplitude of 2 mm in a FAME incubator (Hamilton). TY medium (16 g/L tryptone; 10 g/L yeast extract; 5 g/L NaCl) is used for all cultivations. The cellular growth is monitored by measuring the OD at 600 nm, in 96 well plates as described earlier [38].

The platform is connected to the iLab-Bio database (infoteam Software AG, Bubenreuth, Germany). All generated data and needed set points are stored in and read from this database [39].

### 2.2. Strains, Cell Competence and Transformation

For the manual preparation of competent cells, *E. coli* TG1 (see Table 1 for all strains) was cultivated in 10 ml of TY medium at 37 °C until an OD_600_ of approximately 0.8. Cells were harvested from 200 µL culture by centrifugation, resuspended in 200 µL ice-cold CaCl_2_ solution (100 mM CaCl_2_, 50 mM Tris-HCl pH 7.5), and incubated on ice for 30 min. Finally, the cell pellet was obtained by centrifugation and resuspended in 100 µL ice-cold CaCl_2_ solution and left on ice for at least 2 h.

As no ice is available at the liquid handling platform, the competence protocol was adjusted as follows: 4 °C cold solutions were used and all incubation steps were carried out at 4 °C. Automated cell treatment is described in the results section (Section 3.2, Section 3.3 and Section 3.4).

Manual transformation was carried out as follows: 20 µL competent cells were incubated with 1 ng pUC19 [13] for 30 min, on ice or at 4 °C, respectively. Afterwards, cells were heat shocked for 2 min at 42 °C, 180 µL TY added, shaken at 37 °C for one hour, spread on an agar plate with 125 µg mL^−1^ ampicillin and incubated at 37 °C overnight. For the automated transformation protocol see the results in Section 3.5.

For the determination of the optimal harvesting point for the preparation of competent cells, the *E. coli* strain BW25113 was cultivated in 10 mL TY medium at 37 °C. After 0.5; 1; 1.5; 2; 2.5; 3; 4; 5; 6; and 16 h OD_600_ was measured and equal numbers of cells were harvested (200 µL × 0.8 OD_600_). Competent cells were prepared as above. 50 µL of competent cells were used for transformation with 1 ng pUC19 as described above.

### 2.3. Computational Methods

All computation steps were performed in MATLAB 2016a (Natick, MA, USA). Based on the equation given by Enfors and Häggström 2010 [43] the growth rate is calculated by Equation (1)

(1) $$µ=\frac{ln\left(\frac{X_{k}}{X_{k-1}}\right)}{t_{k}-t_{k-1}}$$

where µ is representing the specific growth rate [h^−1^], *X* the biomass as OD600 and *t* the time [h]. Based on Equation (1), the biomass for the next hour is estimated with

(2) $$X_{k+1}=X_{k}*e^{µ\left(t_{k+1}-t_{k}\right)}$$

The biomass ($X_{k}$) at $t_{k}$ to reach the desired biomass at $t_{k+1}$ (X_Threshold_) is calculated with Equation (3).

(3) $$X_{k}=\frac{X_{threshold}}{e^{µ\left(t_{k+1}-t_{k}\right)}}$$

The dilution factor is calculated with X_k_ from Equation (3) divided by the actual measured biomass.

## 3. Results

The aim of this study is the development of a protocol on the liquid handling platform, to generate competent cells, and transform them directly with one or more plasmids. One of the main problems when transforming different strains with unknown growth characteristics in parallel is the correct harvesting point for all clones. Additionally, this point must be reached at the same time by all cultures to allow running subsequent procedures in parallel.

We compare the quasi-turbidostat method against the traditional batch cultivation to reach the optimal harvesting point. Due to the complexity and fault promptness of integrated high throughput robots, the focus is set on robustness (maximizing the production of competent cells for sufficient transformation). For the development of our method, we used *E. coli* as a case study.

### 3.1. Determination of the Optimal Harvest Conditions

**Figure 3:**
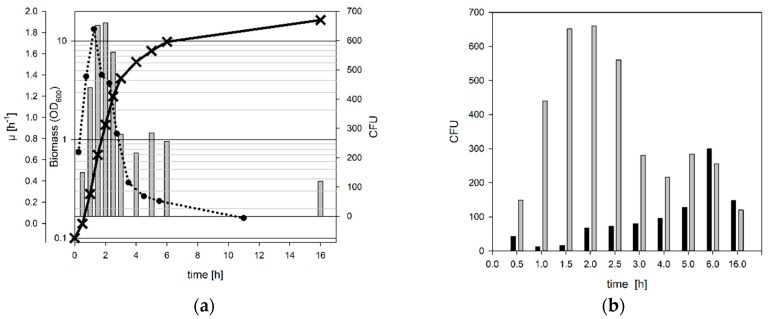
*E. coli* BW25113 was grown in TY medium, equal numbers of cells were harvested at different time points, competent cells were prepared and transformed with 1 ng pUC19 as described in materials and methods. (**a**) Black line: growth curve; dotted line: growth rate [h^−1^]; grey bars: number of transformants. Resulting colonies were counted after overnight incubation at 37 °C. A correlation between transformation efficiency and growth rate can be detected (**b**) grey bars: number of transformants; black bars: number of satellite colonies. Comparison of transformants and satellite colonies obtained after transformation of competent cells as in (**a**). A lower number of satellite colonies indicates higher quality of the competent cells.

The use of highly competent cells facilitates transformation. Due to their high transformation efficiency, low plasmid DNA concentrations or ligation mixtures result in a high number of CFUs. The optimal harvest point for preparation of competent E. coli cells is assumed to be from the early to mid-exponential growth phase [44]; probably the best competence is obtained with cells growing at their maximum specific growth rate. This can also be seen by the number of colonies obtained after transformation of E. coli BW25113 harvested at different growth phases in a typical batch shake flask experiment (Figure 3a). As expected, mid log phase cultures with an OD_600_ between 0.7 and 1.4 proved to be optimal. In contrast, cells from the early or late exponential phase (OD_600_ 0.3 and 2.7) resulted in slightly less colonies and cells from either the lag phase or the stationary phase resulted in significantly less colonies. Interestingly, at the same time the number of satellite colonies resulting from not transformed cells growing in the vicinity of real transformants increased over the growth curve (Figure 3b).

In other words, by creating a system where cells are maintained at maximum growth rate until the harvesting point has been reached, we obtain a highly effective transformation system that is also flexible to cope with the needs of other units, personnel, or sudden faults in the system. As shown in materials and methods, the setpoint for biomass concentration was set to 0.8 to assure no oxygen limitation minimized the cultivation time but also to assure that the cell density was high enough to compensate for low transformation efficiencies. Nevertheless, high transformation efficiencies are not necessary for most applications as for all further steps a few positive transformants are sufficient.

### 3.2. Competent Cells with Batch Cultivation

First, batch cultivations with an adapted sampling were performed. To solve the problem of different lag-phases and stating ODs, precultures were performed for 8 h. Afterwards, the OD_600_ was measured and a new cultivation with fresh medium was prepared. From the precultures, 10 µL were taken by the robot as inoculum for the new main cultivation plate. In this second plate OD_600_ was monitored every hour. If the mean of all cultivations reaches a threshold of 0.4, the monitoring interval was shortened to 30 min. After the mean of all performed cultivations reached a threshold of 0.7, the cells were harvested.

**Figure 4:**
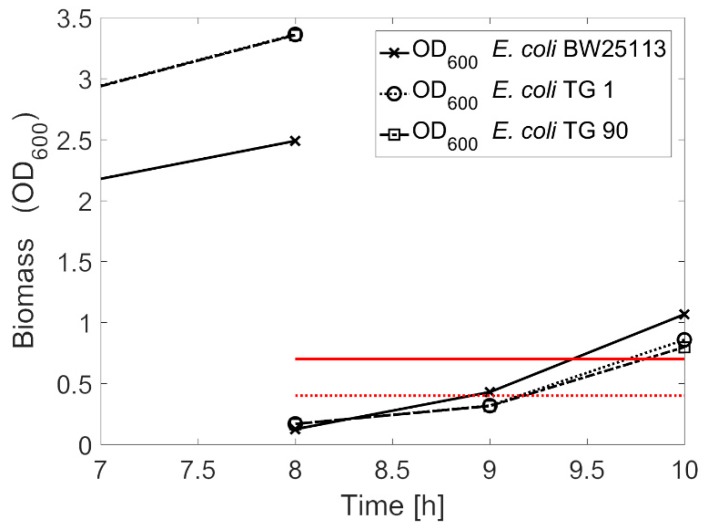
Batch cultivation approach for harvesting of competent cells with Preculture (0–8 h) and a main cultivation (8–10 h). Lower dotted red line: threshold of sampling interval adaptation; upper red line threshold for harvesting. The square of *E. coli* TG 90 is mostly hidden under the circle of *E. coli* TG 1.

Examples for measured OD_600_ values are shown in Figure 4. The OD_600_ after the precultures were 2.50, 2.48, and 3.36 for the *E. coli* strains TG1, TG90, and BW25113, respectively. For the main cultivation, 10 µL of each culture were taken. This corresponds to a 1:20 dilution. After the second measurement, during the main cultivation, the mean OD of all cultures was 0.37 and therefore below the threshold for adapting the sampling mode. Hence, the next sample was taken one hour later. However, the *E. coli* TG1 and TG 90 were already over the threshold of 0.4. For these two strains the sampling point one hour later was already suboptimal. At the third measurement, the *E. coli* strains TG 1 and TG 90 were—with 1.11 and 1.02—out of the optimal range for harvesting, only the *E. coli* BW25113 strain was at the intended point with a mean of 0.82. Since only one of the strains reached an optimal threshold of 0.8 with the batch cultivation method, the adaptation of the experiment to the strains’ growth conditions must have been insufficient. Obviously, the chosen thresholds were not optimal, leading to a suboptimal harvest time. Additionally, this method requires continuous re-tuning, making it time intensive and not proper for a high-throughput system.

### 3.3. Competent Cells in the Quasi-Turbidostat

**Figure 5:**
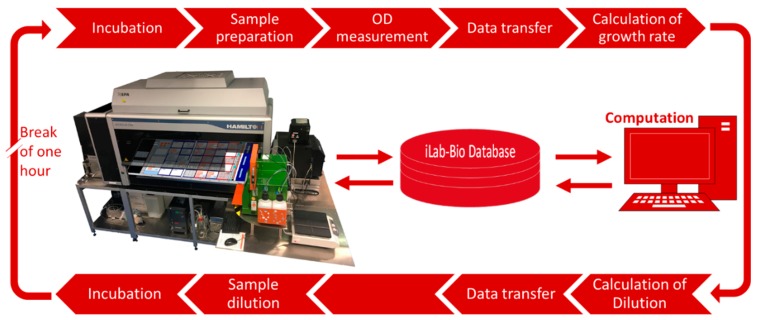
Robotic platform and workflow used for the quasi-turbidostat cultivation for the preparation of competent cells. The incubator is mounted on the left site of the liquid handling station, the plate reader is mounted on the right site. During the quasi-turbidostat cycle, the cells are transferred from the incubator to the liquid handler, a sample is taken, diluted, and measured in the plate reader. Afterwards, the OD_600_ values are transferred into the database and the execution of the script is triggered. The script calculates the current dilution of the quasi-turbidostat cultivations and sends the set points back to the database. Subsequently, the liquid handler reads the set points for the database and executes the dilution step. The cells are incubated for one hour until the next cycle is started.

As mentioned before, the ideal solution to this issue would be a parallel turbidostat system. Therefore, we developed a quasi-turbidostat on a 96-well plate using the liquid handling platform. For the quasi-turbidostat, no preculture is needed. The cultivation is operated in a loop with one-hour cycles containing the following steps (I) sampling, (II) OD_600_ measurement, and (III) dilution (Figure 5).

The first 20 µL sample is taken directly after inoculation, 1:5 diluted, OD_600_ is measured, and the measured values are transferred into the database. Afterwards, the execution of a MATLAB (Mathworks) script is triggered (Source Code S4). During the execution of the script, the program reads the OD_600_ values from the database and the required biomass to reach the targeted OD_600_ in one hour is calculated based on Equation (3). Depending on the calculated OD_600_ values (X_0_), volumes for removing cell suspension and adding fresh medium are sent as setpoints to the database. These setpoints are read out and used by the pipetting robot to perform the dilution step. If no dilution is necessary, only the sampling volume is added to assure a constant cultivation volume of 170 µL over the whole cultivation.

**Figure 6:**
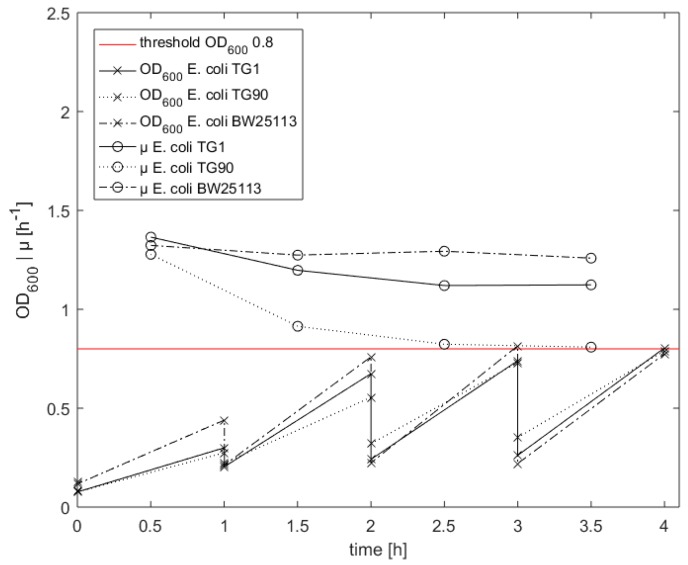
Quasi-turbidostat approach for harvesting of competent cells in a defined physiological state. Biomass is measured every hour. Depending on µ and biomass a dilution of the culture is performed.

In Figure 6, exemplary the OD_600_ measurements of *E. coli* TG1, TG 90, and BW25113 cultures are shown. After the second measurement, the first dilution was calculated. The growth rates at this time were 1.37, 1.28, and 1.32 h^−1^ with OD_600_ values of 0.30, 0.28 and 0.44, respectively. Assuming a constant growth rate, one hour later the OD_600_ was estimated to be 1.17, 0.99, and 1.65 h^−1^, respectively, by applying Equation (2). Therefore, from the beginning, a dilution for all strains was needed. At the third measurement, the OD_600_ of *E. coli* BW15113 was 0.76 and thus very close to the threshold. On the contrary, the growth rates of *E. coli* TG 1 and TG 90 were lower than assumed, as the OD_600_ values of these strains were only 0.68 and 0.56, respectively, and thus clearly below the targeted OD_600_ value. The same behavior is observed at the fourth sampling point after three hours. However, the distances to the target value were lower. The observed OD_600_ values were 0.74 and 0.73 for *E. coli* TG 1 and TG 90, respectively. The *E. coli* BW25513 strain was, with an OD_600_ of 0.82, again the best matching strain. During samplings four and five (hours three to four) all growth rates stayed constant compared to the former interval. The OD values at the last sampling point were 0.80, 0.79, and 0.78 for *E. coli* TG 1, *E. coli* TG 90, and *E. coli* BW25113, respectively. This was a very low deviation from the threshold for all strains and the signal to continue with the cell treatment was given.

### 3.4. Cell Treatment for Competence

For automated cell harvest the cultivation plate was transferred to a position of the 4 °C rack on the liquid handler (Figure 2) to cool down the cells. The whole available culture volume was taken and transferred to a 0.2 µm 96 well filter plate. This filter plate was placed on a vacuum station, integrated on the liquid handler (Figure 2). Over a time of 60 sec, a vacuum of 300 mbar was created to remove the medium from the cells. Afterwards, the cells were resuspended with cold CaCl_2_. This step was repeated three times before transferring the cells into a 96 well PCR plate to enhance the temperature transfer.

### 3.5. Transformation

After the incubation time of two hours at 4 °C, plasmid DNA was dispensed into the incubated cell suspension. The cells were further incubated for 30 min at 4 °C, and subsequently a heat shock at 42 °C for 2 min was performed by moving the PCR plate from the 4 °C rack to the 42 °C rack. And afterwards back to the position on the 4 °C rack. While keeping the cells cooled, a new U-shaped plate with fresh medium was prepared and the whole batches from the PCR plate were transferred into this medium. The U-shaped plate was then cultivated for one hour. Finally, 200 µL of each culture were dispensed on TY agar, which was prepared in 6 well plates. These agar plates were shaken to spread the liquids even over the whole area of the wells. Afterwards, the agar plates were transferred into an incubator and cultivated for at least 12 h at 37 °C.

**Figure 7:**
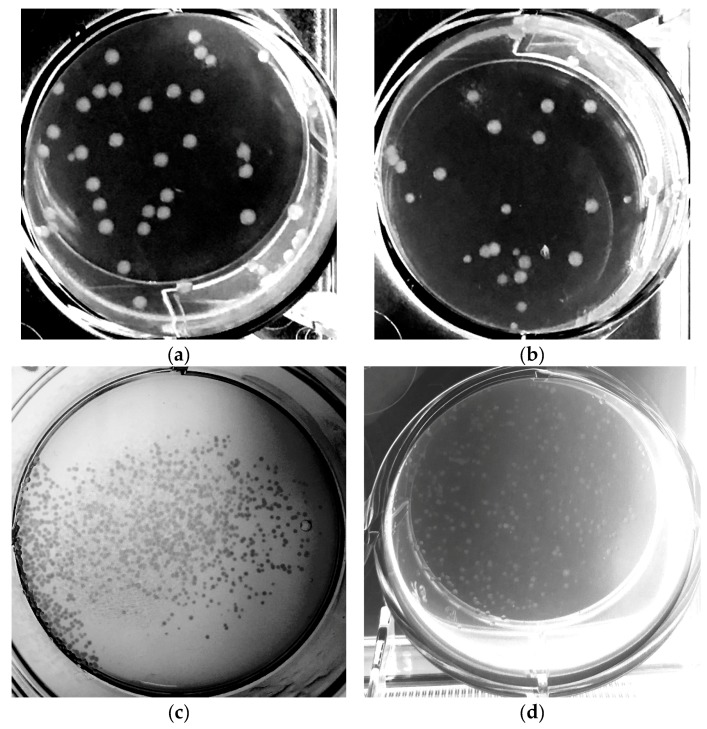
Spread cells after treatment for competence and transformation in 6-well plates (**a**) manually on ice; (**b**) manually at 4 °C instead keeping cell on ice; (**c**) automated treatment with a batch for cell harvesting; (**d**) automated treatment with quasi-turbidostat for cell harvesting.

On all plated wells, the number of observed colonies was high enough for colony picking. In Figure 7, the wells of (a) a manual transformation on ice and (b) with 4° C treatment, (c) the automated treatment with the batch cultivation, and (d) the treatment with the quasi-turbidostat method is shown to be exemplary for the *E. coli* BW25113 strain.

No significant differences were observed by comparing the manual cell treatment on ice with the storage at 4 °C. Both methods lead to 30–50 transformed cells per well and thus we conclude that the use of 4 °C instead of keeping the cells on ice has no negative influence on the transformation efficiency. Moreover, the results show that with the applied shaking protocol the cells are equally spread over the area of the well without the use of a spatula, indicating that shaking the plates only in a one-dimensional movement is sufficient for spreading.

Differences between the automated and manual treatments (comparison of Figure 7b with Figure 7c or Figure 7d) are mainly caused by the different volumes used for plating. Both methods on the liquid handling platform are suitable for the treatment of competent cells and the transformation. Accordingly, as the results of both automated approaches show, there are enough colonies for colony picking. Apart from this, there were no significant differences observed for the *E. coli* BW15113 between the batch treatment and the quasi-turbidostat method. However, the quasi-turbidostat provides a way to guarantee that clones with different specific growth rates are harvested in the growing state at similar cell densities.

## 4. Discussion

The development of an automated method to obtain competent cells when whole strain libraries need to be transformed is a decisive step towards fully automated screening processes. This closes the gap between existing strain and vector libraries and high-throughput screening processes. To our knowledge, this is the first description of a turbidostat implementation in a 96 well plate and therefore, also an important step for the development of advanced screenings and phenotyping applications. The system was tested in an automated robotic facility, so it can be directly included in a broader process development framework reaching up to scale-down experiments at mL scale [45,46]. In order to increase a liquid handling facility towards an automated bioprocess development platform by [47,48], this method can be further connected to automated image analysis, colony counting, and clone picking.

For the development of this method we compared two different strategies for cell harvesting, a classical batch approach and a novel quasi-turbidostat approach. With the latter one we were able to harvest strains with different growth characteristics at the best harvesting point (i.e., cell density and growth stage), independent from the source of the strains; e.g., an agar plate, cryo-stock or another liquid culture.

The batch approach is simple to implement, but due to the different growth characteristics of the hosts, changing harvesting points, sensitivity to faults in the system as well as initial concentrations, and low time flexibility it is not suitable for use in a high throughput.

The use of the quasi-turbidostat provides some important advantages compared to the batch method. The sampling point for all strains was reached perfectly after four hours. The only adjustable parameter is the cycle time, which can automatically be adjusted to each well for a wide coverage of fast and slow growing cells. In the case study, the cycle time was one hour, therefore a minimal growth rate of 0.1 h^−1^ is needed, but an automated adjustment of the cycle time, to cover also slower and faster growing cells is straight forward.

However, the samples had to be diluted already after the first analytical cycle. Therefore, we expected to reach the threshold for all strains directly after the first cycle. As shown above (Figure 2b), this was not the case. Our method requires a constant growth rate to get optimal results. During the first two cycles, until hour three, we saw a decrease in the growth rate of the *E. coli* strains TG1 and TG90 mainly caused by inaccuracies of the OD measurement at low values. Our method calculated the dilutions with an unprecise µ and therefore reached suboptimal results. Whether the decrease in the growth rate is caused by measurement errors or by a physiological background could be in the focus of further investigations. It is known that acetic acid has a negative influence on the glucose uptake rate at high levels and therefore is known to reduce the maximum specific growth rate [45,49]. Hence, the acetic acid concentration is also diluted in every cycle and should not reach a critical concentration. It has been reported that the maximum glucose uptake rate decreases significantly over the time in glucose limited fed-batch cultivation with a constant feed profile: i.e., at lower specific growth rates [50,51].

To ensure a sufficient transformation efficiency especially when using low plasmid concentrations or ligation mixtures, it is important to harvest all cells during the optimal growth phase. The benefit of the quasi-turbidostat compared to the batch method is that the former ensures this because it keeps strains with different growth rates in the logarithmic growth phase until all cells reach the optimal harvesting point for the preparation of competent cells and their subsequent transformation.

To maximize our throughput and keeping the system as simple as possible, we used 96 well plates for cultivation and cell treatment. The use of enhanced high throughput cultivation systems like minibioreactor systems (MBRs) [52] could be alternatively considered if higher titers are required, and applications based on the online biomass signal have already been reported [33]. Nevertheless, this would increase the complexity of the system. Apart from that, our developed quasi-turbidostat method could be adapted to MBR with online biomass monitoring. Other systems that are very well suited for the quasi-turbidostat method are: BioLector [53], m2p, HEL minireactors, etc.

In addition, the development of a high-throughput method protocol for competent cell treatment and transformation, also describes a method that does neither need ice nor centrifugation. In contrast to the manual preparation of competent cells where centrifugation is used for harvesting and washing steps, no centrifuge is needed for the automated protocol. Also, filtration has several advantages compared to centrifugation; (1) washing steps are more accurate as the media can be removed thoroughly without touching the cell pellet; (2) a vacuum station can be easier integrated in a liquid handling station as it needs less space and no hardware modifications and thus (3) investment costs are significantly lower.

The protocol is also very useful for laboratories with limited equipment or for a parallel treatment of cells manually.

## 5. Conclusions

The developed method is able to treat *E. coli* cells for competence and transform them with a certain vector and could be adapted to other strains with a similar protocol.

As show in Figure 4, the results are good enough to transfer the cells for further colony picking. Our automated method ended with the incubation of the spread agar plates. The created strain library is the basis of further automated screening and strain engineering methods [46,54]. Furthermore, the method could be adapted to other organisms.

## Figures and Tables

**Table 1:** List of used strains.

Strain	Genotype	Source
*E. coli* TG 1	K-12 *supE hsd Δ5thi Δ[lac-proAB]* F′ *[traD36 proAB lacIq lacZ ΔM15]*	[40]
*E. coli* TG 90	K-12 *supE hsd Δ5thi Δ[lac-proAB]* F′ *[traD36 proAB lacIq lacZ ΔM15] *pcnB80* zad*::*TnlO*	[41]
*E. coli* BW25113	K-12 *lacI*^+^ *rrnB*_T14_ Δ*lacZ*_WJ16_ *hsdR*514 Δ*araBAD*_AH33_ Δ*rhaBAD*_LD78_ *rph-1* Δ*(araB–D)567* Δ*(rhaD–B)568* Δ*lacZ4787*(::*rrnB-3*) *hsdR514 rph-1*	[42]

## Supplementary Materials

The following are available online at http://www.mdpi.com/2076-2607/6/3/60/s1, Source Code S1: Hamilton Script Overview, Source Code S2: Hamilton Script detail, Figure S3: Tecan LHS, Source Code S4: MATLAB Source Code.
