# Supplementary material for: Automated Cell Treatment for Competence and Transformation of Escherichia coli in a High-Throughput Quasi-Turbidostat Using Microtiter Plates
Source: Microorganisms. 2018 Jun 25;6(3):60. doi: 10.3390/microorganisms6030060 (PMC6163857; doi:10.3390/microorganisms6030060)
Supplement: Supplementary file 1 [file microorganisms-06-00060-s001.pdf]

# Automated cell treatment for competence and transformation of *Escherichia coli* in high throughput

**Sebastian Hans, Mathias Gimpel, Florian Glauche, Peter Neubauer and M. Nicolas Cruz-Bournazou ***

Technische Universität Berlin, Institute of Biotechnology, Chair of Bioprocess Engineering, Ackerstraße 76, D-13357 Berlin, Germany

* Corresponding author: mariano.n.cruzbournazou@tu-berlin.de; Tel.: +49-30-314-72626

## Inhalt

Source Code S1: Hamilton Script Overview .......... 1

Source Code S2: Hamilton Script detail .......... 2

Figure S3: Tecan LHS .......... 7

Source Code S4 MATLAB Source Code .......... 8

## Source Code S1: Hamilton Script Overview

| | Method |
|---|---|
| 1 | Assignment<br>'main_runID' = '325' |
| 2 | HSL code.<br>main_current_date_str = TimGetFormattedDate("%Y-%m-%d") + "_" + TimGetFormattedTime("%H_%M_%S"); |
| 3 | Assignment<br>'main_cultivation_dilution' = '10' |
| 4 | Assignment<br>'main_cultivation_cycletime_min' = '60' |
| 5 | Assignment<br>'main_incubation_onIce' = '1800' |
| 6 | Assignment<br>'main_Operator_Number' = "'491775724287'" |
| 7 | Grouping<br>Konstanten |
| 50 | Grouping<br>Initialize |
| 61 | Grouping<br>Cultivation |
| 138 | Grouping<br>Filtrieren |
| 150 | Grouping<br>Resuspendiren |
| 159 | Grouping<br>Incubation on ice |
| 164 | Grouping<br>Transformation |
| 174 | Grouping<br>Incubation on Ice &<br>Prepair fresh media plate |
| 181 | Grouping<br>Heatshock |
| 189 | Grouping<br>Cultivation2 |
| 198 | Grouping<br>Ausplatieren |
| 231 | |

# Source Code S2: Hamilton Script detail

| | Method |
|---|---|
| 1 | Assignment<br>'main_runID' = '325' |
| 2 | HSL code.<br>main_current_date_str = TimGetFormattedDate("%Y-%m-%d") + "_" +<br>TimGetFormattedTime("%H_%M_%S"); |
| 3 | Assignment<br>'main_cultivation_dilution' = '10' |
| 4 | Assignment<br>'main_cultivation_cycletime_min' = '60' |
| 5 | Assignment<br>'main_incubation_onIce' = '1800' |
| 6 | Assignment<br>'main_Operator_Number' = '"4917757 24287"' |
| 7 | Grouping<br>Konstanten |
| 8 | Assignment<br>'main_Gen5_PlateCounter' = '2' |
| 9 | Assignment<br>'main_PreCulture_KryoVol' = '5' |
| 10 | Comment<br><main_PreCulture_IncubationTime in Hours!> |
| 11 | Array: Declare / Set Size<br>Set array 'main_PreCulture_IncubationTime' to empty size. |
| 12 | Assignment<br>'main_PreCulture_dilution' = '10' |
| 13 | Assignment<br>'main_cultivationVol' = '170' |
| 14 | HSL code.<br>// Berechne Preculture Media Volume<br>main_PreCulture_MediaVol = main_cultivationVol - main_PreCulture_KryoVol;<br><br>// Teile PreCulte Cultivation Time in 3 Intervalle ein (1 /3,5 / 3,5 = 8)<br>// Umrechnen der Kultivierungszeit von Stunden in Sekunden<br>main_PreCulture_IncubationTime.AddAsLast(3600);<br>main_PreCulture_IncubationTime.AddAsLast(3600);<br>main_PreCulture_IncubationTime.AddAsLast(3600);<br>main_PreCulture_IncubationTime.AddAsLast(3600);<br>main_PreCulture_IncubationTime.AddAsLast(3600);<br>main_PreCulture_IncubationTime.AddAsLast(3600);<br>main_PreCulture_IncubationTime.AddAsLast(3600);<br>main_PreCulture_IncubationTime.AddAsLast(3600);<br>main_PreCulture_IncubationTime_temp = 0;<br><br>main_PreCulture_LoopNum = main_PreCulture_IncubationTime.GetSize();<br><br>// Rechne Verdünnungen für die ODMessung aus<br>main_PreCulture_VolSample = main_cultivationVol / main_PreCulture_dilution;<br>main_PreCulture_VolNaCl = main_cultivationVol - main_PreCulture_VolSample; |
| 15 | Assignment<br>'main_reader_RowName' = '"OD600"' |
| 16 | Array: Declare / Set Size<br>Set array 'main_reader_PreCultureValues_1' to empty size. |
| 17 | Array: Declare / Set Size<br>Set array 'main_reader_PreCultureValues_2' to empty size. |
| 18 | Array: Declare / Set Size<br>Set array 'main_reader_PreCultureValues_3' to empty size. |
| 19 | Assignment<br>'main_OD600_threshold' = '0,041' |
| 20 | Assignment<br>'main_OD600_correctionFactor' = '2' |



| | Method |
|---|---|
| 21 | Assignment<br>'main_cultivation_VolPreCulture' = '10' |
| 22 | HSL code.<br>main_cultivation_VolMedium = main_cultivationVol - main_cultivation_VolPreCulture; |
| 23 | Assignment<br>'main_cultivation_VolSample' = '20' |
| 24 | HSL code.<br>main_cultivation_VolNaCl = 200 - main_cultivation_VolSample; |
| 25 | Assignment<br>'main_cultivation_slot' = '4' |
| 26 | Assignment<br>'main_cultivation_temp' = '37' |
| 27 | Assignment<br>'main_cultivation_speed' = '1000' |
| 28 | Assignment<br>'main_reader_path2resultsFolder' = '"C:\\Dokumente und Einstellungen\\Peter Neubauer\\Eigene Dateien\\Sebastian\\Robot"' |
| 29 | Assignment<br>'main_reader_path2experimentFolder' = '"C:\\Programme\\BioTek\\Gen5 1.09\\Experiments\\Sebastian\\Robot"' |
| 30 | HSL code.<br>Shell("C:\\WINDOWS\\system32\\cmd.exe /C mkdir \"" + main_reader_path2resultsFolder +<br>"\\" + main_current_date_str + "\"", 2, 1);<br>Shell("C:\\WINDOWS\\system32\\cmd.exe /C mkdir \"" +<br>main_reader_path2experimentFolder + "\\" + main_current_date_str+ "\"", 2, 1);<br>Shell("C:\\WINDOWS\\system32\\cmd.exe /C copy \"" +<br>main_reader_path2experimentFolder + "\\" + "_default_OD600.xpt\"" + " \"" +<br>main_reader_path2experimentFolder + "\\" + main_current_date_str + "\\" + |
| 31 | Assignment<br>'main_cultivation_abort' = '0' |
| 32 | Assignment<br>'main_filter_VolFromCultur' = '180' |
| 33 | HSL code.<br>// Rechne Cycletime in Sec<br>main_cultivation_cycletime_sec = main_cultivation_cycletime_min * 60;<br><br>// Ermittle wieviele Stämme vonhanden sind<br>main_numStrains = ML_STAR._Res_Kryos.GetTotal(); |
| 34 | Assignment<br>'main_pump_ID' = '1' |
| 35 | Assignment<br>'main_pump_COMPort' = '3' |
| 36 | Assignment<br>'main_pump_dPressure' = '200' |
| 37 | Assignment<br>'main_pump_tresholdPressure' = '10' |
| 38 | Assignment<br>'main_filter_duration' = '20' |
| 39 | Assignment<br>'main_wash_Volume' = '200' |
| 40 | Assignment<br>'main_wash_cycles' = '3' |
| 41 | Assignment<br>'main_resuspend_Vol' = '150' |
| 42 | Assignment<br>'main_incubation_time1' = '7200' |
| 43 | Assignment<br>'main_dna_Vol' = '2' |

| | Method |
|---|---|
| 44 | Assignment<br>'main_incubation_heatShock' = '120' |
| 45 | Assignment<br>'main_cultivation2_Vol' = '150' |
| 46 | HSL code.<br>main_cultivaiton2_VolKultur = 200 - main_cultivation2_Vol; |
| 47 | Assignment<br>'main_cultivation2_time' = '3600' |
| 48 | Assignment<br>'main_plating_vol' = '200' |
| 49 | Grouping |
| 50 | Grouping<br>Initialize |
| 51 | If, Else<br>(main_runID is NOT equal to 0) |
| 52 | iLAB_connect of BVTlib_ilab_connector<br>iLAB_connect() |
| 53 | getBioreactorIDs of BVTlib_ilab_connector<br>main_bioID_array = getBioreactorIDs(main_runID) |
| 54 | Assignment<br>'main_bioID' = 'main_bioID_array[1]' |
| 55 | End If |
| 56 | HSL code.<br>main_StatusLTU = MessageBox("Is it Plate of the LTU on the site of the Hamilton?","Status LTU", 4);<br>if (main_StatusLTU == 6) {<br>Position_Tecan();<br>} |
| 57 | Initialize (Single Step) on ML_STAR<br>Always initialize: Off<br>3 return value(s) . |
| 58 | initialize_Fame of unifiedlibrary<br>UNIFIEDLIBRARY::initialize_Fame(ML_STAR, main_cultivation_temp, 2, main_cultivation_speed, ML_STAR._Plate_Cultivation) |
| 59 | BVSInitialize of HSLVacuuBrandPump<br>HSLStarBVSLib::BVSInitialize(main_pump_ID, main_pump_COMPort) |
| 60 | Grouping |
| 61 | Grouping<br>Cultivation |
| 62 | Comment<br><Prepair PreCulture Plate> |
| 63 | Sub_01_AddFromContainer_sterilTips of KompetenteZellen_v0.0.1<br>Sub_01_AddFromContainer_sterilTips(ML_STAR._Res_Medium, ML_STAR._Plate_Preculture, main_PreCulture_MediaVol) |
| 64 | Sub_02_AddFromSequence of KompetenteZellen_v0.0.1<br>Sub_02_AddFromSequence(ML_STAR._Res_Kryos, ML_STAR._Plate_Preculture, main_PreCulture_KryoVol) |
| 65 | Comment<br><Start Preculture> |
| 66 | fame_transport of unifiedlibrary<br>UNIFIEDLIBRARY::fame_transport(2, ML_STAR._Plate_Preculture, main_cultivation_slot, main_cultivation_temp, main_cultivation_speed, ML_STAR) |
| 67 | Timer: Start<br>Start timer 'main_timer_preculture', set to relative time: '0' [s] |



| | Method |
|---|---|
| 68 | Cleaning_0050_Tips of SM_SH_Cleanning<br>SM_SH_CLEANNING::Cleaning_0050_Tips(ML_STAR, ML_STAR._Tips_0050_1, ML_STAR._Res_EtOH, ML_STAR._Res_EtOH_Lid, ML_STAR._Res_EtOH_Lid_Storage, |
| 69 | sendWhatsApp of SM_Gluocse<br>SM_GLUOCSE::sendWhatsApp(main_Operator_Number, "First Timer is started ...") |
| 70 | Loop<br>'main_PreCulture_LoopNum' times<br>'main_PreCulture_Loop' used as loop counter variable |
| 71 | Grouping<br>Timer |
| 72 | Timer: Wait for<br>Wait for timer 'main_timer_preculture', showtimer display, is stoppable |
| 73 | Timer: Start<br>Start timer 'main_timer_preculture', set to relative time: 'main_PreCulture_IncubationTime[main_PreCulture_Loop]' [s] |
| 74 | sendWhatsApp of SM_Gluocse<br>SM_GLUOCSE::sendWhatsApp(main_Operator_Number, "Next Timer is started ...") |
| 75 | Grouping |
| 76 | Comment<br><Take newPlate> |
| 77 | 1000µl Channel CO-RE Grip Get Plate (Single Step) on ML_STAR<br>Transport mode: (0) Plate only, Sequence: ML_STAR._Plate_Storage, Sequence counting: (1) Automatic, Channel to be used: 8<br>3 return value(s) . |
| 78 | 1000µl Channel CO-RE Grip Place Plate (Single Step) on ML_STAR<br>Transport mode: (0) Plate only, Sequence: ML_STAR._Plate_working, Sequence counting: (0) Manually, Eject tool: (1) Yes<br>3 return value(s) . |
| 79 | Sub_01_AddFromContainer of KompetenteZellen_v0.0.1<br>Sub_01_AddFromContainer(ML_STAR._Res_NaCl, ML_STAR._Plate_working, main_PreCulture_VolNaCl) |
| 80 | Comment<br><Hole Platte aus dem Inkubator> |
| 81 | fame_transport of unifiedlibrary<br>UNIFIEDLIBRARY:: fame_transport(1, ML_STAR._Plate_FromIncubator, main_cultivation_slot, main_cultivation_temp, main_cultivation_speed, ML_STAR) |
| 82 | Comment<br><Messe OD> |
| 83 | Sub_02_AddFromSequence_96 of KompetenteZellen_v1.1.0<br>Sub_02_AddFromSequence_96(ML_STAR._Plate_FromIncubator, ML_STAR._Plate_working, main_PreCulture_VolSample) |
| 84 | Comment<br><Lege Platte zurück in den Inkubator> |
| 85 | fame_transport of unifiedlibrary<br>UNIFIEDLIBRARY:: fame_transport(2, ML_STAR._Plate_FromIncubator, main_cultivation_slot, main_cultivation_temp, main_cultivation_speed, ML_STAR) |
| 86 | Grouping<br>ReaderMessung und Daten erreichnen |

| | Method |
|---|---|
| 87 | HSL code.<br>// Erstelle Path zum Experiment<br>main_reader_path2experiment = main_reader_path2experimentFolder + "\\"<br>+ main_current_date_str + "\\" + main_current_date_str + "_OD600.xpt";<br>// Erstelle Phat zur Exceldatei mit den Ergebnissen<br>main_reader_path2results = main_reader_path2resultsFolder + "\\" +<br>main_current_date_str;<br><br>main_reader_filenameResults = main_current_date_str +<br>"_OD600_Preculture_" + IStr(main_PreCulture_Loop);<br>main_reader_TabName = "Plate " + IStr(main_Gen5_PlateCounter) + " -<br>Data";<br>main_Gen5_PlateCounter++;<br>main_reader_filenameReturn = "C:\\Dokumente und Einstellungen\\Peter<br>Neubauer\\Eigene Dateien\\Sebastian\\Robot\\" + main_current_date_str +<br>"\\" + main_reader_filenameResults + ".xls"; |
| 88 | Readermessung of unifiedlibrary<br>UNIFIEDLIBRARY::Readermessung(ML_STAR, HxGen5,<br>ML_STAR._Plate_working, main_reader_path2experiment,<br>main_reader_path2results, main_reader_filenameResults,<br>main_cultivation_dilution, main_cultivation_start,<br>main_reader_filenameReturn) |
| 89 | Comment<br><Werte OD aus> |
| 90 | readRow of BVTlib_ExcelImport<br>main_reader_resultes = readRow(main_reader_filenameReturn,<br>main_reader_TabName, main_reader_RowName) |
| 91 | subArray of BVTlib_ExcelImport<br>main_reader_resultesSub = subArray(main_reader_resultes, 1,<br>main_numStrains) |
| 92 | subtractArray of BVTlib_ExcelImport<br>main_reader_resultesSub = subtractArray(main_reader_resultesSub,<br>main_OD600_threshold) |
| 93 | multiplyArray of BVTlib_ExcelImport<br>main_reader_resultesSub = multiplyArray(main_reader_resultesSub,<br>main_PreCulture_dilution) |
| 94 | multiplyArray of BVTlib_ExcelImport<br>main_reader_PreCultureValues = multiplyArray(main_reader_resultesSub,<br>main_OD600_correctionFactor) |
| 95 | HSL code.<br>main_TimeStamp_ilab = TimGetFormattedDate("%Y-%m-%d") + " " +<br>TimGetFormattedTime("%H:%M:%S"); |
| 96 | If, Else<br>(main_runID is NOT equal to 0) |
| 97 | sendData_long of BVTlib_ilab_connector<br>sendData_long(main_runID, main_bioID, "OD600",<br>main_reader_PreCultureValues, 1, main_TimeStamp_ilab,<br>main_PreCulture_dilution) |
| 98 | End If |
| 99 | arrayMinValue of BVTlib_ExcelImport<br>main_ODmin = arrayMinValue(main_reader_PreCultureValues) |
| 100 | If, Else<br>(main_ODmin is greater than OR equal to 0,75) |
| 101 | Loop: Break |
| 102 | End If |
| 103 | Grouping |
| 104 | Grouping<br>performing dilution |



| | Method |
|---|---|
| 105 | If, Else<br>(main_runID is NOT equal to 0) |
| 128 | Grouping |
| 129 | Comment<br><Plate2waste> |
| 130 | iSWAP Transport on ML_STAR<br>Transport labware from 'ML_STAR._Plate_working' to 'ML_STAR.platewaste'<br>1 return value(s) . |
| 131 | Cleaning_0050_Tips of SM_SH_Cleanning<br>SM_SH_CLEANNING::Cleaning_0050_Tips(ML_STAR, ML_STAR._Tips_0050_1,<br>ML_STAR._Res_EtOH, ML_STAR._Res_EtOH_Lid,<br>ML_STAR._Res_EtOH_Lid_Storage, 1, 0) |
| 132 | Cleaning_0300_Tips of SM_SH_Cleanning<br>SM_SH_CLEANNING::Cleaning_0300_Tips(ML_STAR, ML_STAR._Tips_0300_1,<br>ML_STAR._Res_EtOH, ML_STAR._Res_EtOH_Lid,<br>ML_STAR._Res_EtOH_Lid_Storage, 0, 1) |
| 133 | End Loop |
| 134 | Comment<br><Hole Plate zurück aus dem Inkubator> |
| 135 | fame_transport of unifiedlibrary<br>UNIFIEDLIBRARY::fame_transport(1, ML_STAR._Plate_FromIncubator,<br>main_cultivation_slot, main_cultivation_temp, main_cultivation_speed, ML_STAR) |
| 136 | sendWhatsApp of SM_Gluocse<br>SM_GLUOCSE::sendWhatsApp(main_Operator_Number, "Cultivation ended") |
| 137 | Grouping |
| 138 | Grouping<br>Filtrieren |
| 139 | Comment<br><Start filtration> |
| 140 | Sub_02_AddFromSequence_300 of KompetenteZellen_v0.0.1<br>Sub_02_AddFromSequence_300(ML_STAR._Plate_FromIncubator,<br>ML_STAR._Plate_Filter, main_filter_VolFromCultur) |
| 141 | ReqActualPressure of HSLVacuuBrandPump<br>main_temp = HSLVacuuBrandPump::ReqActualPressure(main_pump_ID) |
| 142 | BVSVacuum of HSLVacuuBrandPump<br>HSLStarBVSLib::BVSVacuum(main_pump_ID, main_pump_dPressure,<br>main_filter_duration, 1, main_pump_tresholdPressure, main_pump_returnPressure) |
| 143 | Loop<br>'main_wash_cycles' times<br>'main_Loop_Washing' used as loop counter variable |
| 144 | HSL code.<br>Trace("CaCl - Loop");<br>ML_STAR._Plate_Filter.SetCurrentPosition(0); |
| 145 | Sub_01_AddFromContainer of KompetenteZellen_v0.0.1<br>Sub_01_AddFromContainer(ML_STAR._Res_CaCl2, ML_STAR._Plate_Filter,<br>main_wash_Volume) |
| 146 | BVSVacuum of HSLVacuuBrandPump<br>HSLStarBVSLib::BVSVacuum(main_pump_ID, main_pump_dPressure,<br>main_filter_duration, 1, main_pump_tresholdPressure, main_pump_returnPressure) |
| 147 | End Loop |
| 148 | BVSTerminate of HSLVacuuBrandPump<br>HSLStarBVSLib::BVSTerminate(main_pump_ID) |
| 149 | Grouping |
| 150 | Grouping<br>Resuspendiren |

| | Method |
|---|---|
| 151 | HSL code.<br>Trace("Resupend - Loop");<br>ML_STAR._Plate_Filter.SetCurrentPosition(1);<br>main_resuspend_LoopCounter = main_numStrains/8; |
| 152 | Loop<br>'main_resuspend_LoopCounter' times<br>'main_Loop_resuspend' used as loop counter variable |
| 153 | 1000µl Channel Aspirate on ML_STAR<br>Sequence: ML_STAR._Res_CaCl2, Volume [µl]: main_resuspend_Vol<br>0 return value(s) . |
| 154 | 1000µl Channel Dispense on ML_STAR<br>Sequence: ML_STAR._Plate_Filter, Volume [µl]: main_resuspend_Vol<br>0 return value(s) . |
| 155 | 1000µl Channel Aspirate on ML_STAR<br>Sequence: ML_STAR._Plate_Filter, Volume [µl]: main_resuspend_Vol<br>0 return value(s) . |
| 156 | 1000µl Channel Dispense on ML_STAR<br>Sequence: ML_STAR._Plate_PCR, Volume [µl]: Remaining volume inclusive blowout air<br>0 return value(s) . |
| 157 | End Loop |
| 158 | Grouping |
| 159 | Grouping<br>Incubation on ice |
| 160 | Timer: Start<br>Start timer 'main_timer_incutation', set to relative time: 'main_incubation_time1' [s] |
| 161 | iSWAP Transport on ML_STAR<br>Transport labware from 'ML_STAR._Plate_FromIncubator' to 'ML_STAR.platewaste'<br>1 return value(s) . |
| 162 | Timer: Wait for<br>Wait for timer 'main_timer_incutation', show timer display, is stoppable timer. |
| 163 | Grouping |
| 164 | Grouping<br>Transformation |
| 165 | HSL code.<br>Trace("Starting Loop - Transformation");<br>ML_STAR._Plate_PCR.SetCurrentPosition(1); |
| 166 | _Sub05_TransportLid of KompetenteZellen_v0.0.1<br>_Sub05_TransportLid(ML_STAR._Res_Plasmid_Lid, ML_STAR._LidStorage) |
| 167 | Loop<br>'main_resuspend_LoopCounter' times<br>'main_Loop_DNA' used as loop counter variable |
| 168 | 1000µl Channel Aspirate on ML_STAR<br>Sequence: ML_STAR._Res_Plasmid, Volume [µl]: main_dna_Vol<br>0 return value(s) . |
| 169 | 1000µl Channel Dispense on ML_STAR<br>Sequence: ML_STAR._Plate_PCR, Volume [µl]: Remaining volume inclusive blowout air<br>0 return value(s) . |
| 170 | End Loop |
| 171 | _Sub05_TransportLid of KompetenteZellen_v0.0.1<br>_Sub05_TransportLid(ML_STAR._LidStorage, ML_STAR._Res_Plasmid_Lid) |
| 172 | HSL code.<br>Trace("Transformation done"); |
| 173 | Grouping |



| | Method |
|---|---|
| 174 | Grouping<br>Incubation on Ice &<br>Prepair fresh media plate |
| 175 | HSL code.<br>Trace("Incubation on ice"); |
| 176 | Timer: Start<br>Start timer 'main_timer_incutationOnIce', set to relative time: 'main_incubation_onIce' [s] |
| 177 | Sub_01_AddFromContainer_sterilTips of KompetenteZellen_v0.0.1<br>Sub_01_AddFromContainer_sterilTips(ML_STAR._Res_Medium,<br>ML_STAR._Plate_Cultivaiton_2, main_cultivation2_Vol) |
| 178 | Timer: Wait for<br>Wait for timer 'main_timer_incutationOnIce', show timer display, is stoppable timer. |
| 179 | HSL code.<br>Trace("Incubation on ice done"); |
| 180 | Grouping |
| 181 | Grouping<br>Heatshock |
| 182 | HSL code.<br>Trace("Starting Heatshock"); |
| 183 | iSWAP Transport on ML_STAR<br>Transport labware from 'ML_STAR._Plate_PCR' to 'ML_STAR._Plate_PCR_heatshock'<br>1 return value(s) . |
| 184 | Timer: Start<br>Start timer 'main_timer_incutationHeatshock', set to relative time:<br>'main_incubation_heatShock' [s] |
| 185 | Timer: Wait for<br>Wait for timer 'main_timer_incutationHeatshock', show timer display, is stoppable timer. |
| 186 | iSWAP Transport on ML_STAR<br>Transport labware from 'ML_STAR._Plate_PCR_heatshock' to 'ML_STAR._Plate_PCR'<br>1 return value(s) . |
| 187 | HSL code.<br>Trace("Heatshock Done"); |
| 188 | Grouping |
| 189 | Grouping<br>Cultivation2 |
| 190 | HSL code.<br>Trace("Starting Cultivation 2"); |
| 191 | Sub_02_AddFromSequence of KompetenteZellen_v0.0.1<br>Sub_02_AddFromSequence(ML_STAR._Plate_PCR, ML_STAR._Plate_Cultivaiton_2,<br>main_cultivaiton2_VolKultur) |
| 192 | fame_transport of unifiedlibrary<br>UNIFIEDLIBRARY::fame_transport(2, ML_STAR._Plate_Cultivaiton_2,<br>main_cultivation_slot, main_cultivation_temp, main_cultivation_speed, ML_STAR) |
| 193 | Timer: Start<br>Start timer 'main_timer_cultivation2', set to relative time: 'main_cultivation2_time' [s] |
| 194 | Timer: Wait for<br>Wait for timer 'main_timer_cultivation2', show timer display, is stoppable timer. |
| 195 | fame_transport of unifiedlibrary<br>UNIFIEDLIBRARY::fame_transport(1, ML_STAR._Plate_FromIncubator,<br>main_cultivation_slot, main_cultivation_temp, main_cultivation_speed, ML_STAR) |
| 196 | HSL code.<br>Trace("Cultivation 2 Done"); |
| 197 | Grouping |
| 198 | Grouping<br>Ausplatieren |

| | Method |
|---|---|
| 199 | Sequence: Set Current Position<br>current position of sequence 'ML_STAR._Plate_Cultivaiton_2' = '1' |
| 200 | HSL code.<br>Trace("Start Plating");<br>main_temp = 0;<br><br>// Errechne wie viele Platen ausplattiert werden muessen<br>main_platting_numPlates = main_numStrains%6; |
| 201 | Assignment<br>'main_CytomatPos' = '148' |
| 202 | Loop<br>'main_platting_numPlates' times<br>'main_loop_platting' used as loop counter variable |
| 203 | Grouping<br>Get Plate from cytomat |
| 204 | HSL code.<br>sShellCmd = "C:\Dokumente und Einstellungen\Peter Neubauer\Eigene Dateien\Sebastian\Programms\Remote_Trick\ClientConsole.exe C:\Users\BVT-Administrator\Anaconda3\python.exe C:\Tecan_Scripts\Remoting\CytomatAgarplate.py -m 1 -s " + IStr(main_CytomatPos);<br>Trace(sShellCmd);<br>Shell(sShellCmd, 2,1); |
| 205 | Position_Hamilton of BVTlib_LTU<br>Position_Hamilton() |
| 206 | iSWAP Transport on ML_STAR<br>Transport labware from 'ML_STAR._move_Tecan' to 'ML_STAR._Plate_AgarTransport'<br>1 return value(s) . |
| 207 | Grouping |
| 208 | Sequence: Set Current Position<br>current position of sequence 'ML_STAR._LidAgar' = '1' |
| 209 | _Sub05_TransportLid_Agar of KompetenteZellen_v0.0.1<br>_Sub05_TransportLid_Agar(ML_STAR._Plate_Agar_StorePlated_Lids, ML_STAR._LidAgar) |
| 210 | 1000µl Channel CO-RE Grip Get Plate (Single Step) on ML_STAR<br>Transport mode: (0) Plate only, Sequence: ML_STAR._Plate_Agar_StorePlated, Sequence counting: (0) Manually, Channel to be used: 8<br>3 return value(s) . |
| 211 | 1000µl Channel CO-RE Grip Place Plate (Single Step) on ML_STAR<br>Transport mode: (0) Plate only, Sequence: ML_STAR._Plate_Agar, Sequence counting: (0) Manually, Eject tool: (0) No<br>3 return value(s) . |
| 212 | 1000µl Channel Aspirate on ML_STAR<br>Sequence: ML_STAR._Res_Medium, Volume [µl]: 190<br>0 return value(s) . |
| 213 | 1000µl Channel Aspirate on ML_STAR<br>Sequence: ML_STAR._Res_Medium, Volume [µl]: 10<br>0 return value(s) . |
| 214 | 1000µl Channel Aspirate on ML_STAR<br>Sequence: ML_STAR._Plate_FromIncubator, Volume [µl]: 50<br>0 return value(s) . |
| 215 | 1000µl Channel Dispense on ML_STAR<br>Sequence: ML_STAR._Plate_Agar, Volume [µl]: Remaining volume inclusive blowout air<br>0 return value(s) . |
| 216 | Sub_05_Shaking of KompetenteZellen_v0.0.1<br>Sub_05_Shaking() |
| 217 | Sequence: Set Current Position<br>current position of sequence 'ML_STAR._Plate_Agar' = '1' |



| | Method |
|---|---|
| 218 | 1000µl Channel CO-RE Grip Get Plate (Single Step) on ML_STAR<br>Transport mode: (0) Plate only, Sequence: ML_STAR._Plate_Agar, Sequence counting: (0) Manually, Channel to be used: 8<br>3 return value(s) . |
| 219 | 1000µl Channel CO-RE Grip Place Plate (Single Step) on ML_STAR<br>Transport mode: (0) Plate only, Sequence: ML_STAR._Plate_Agar_StorePlated, Sequence counting: (0) Manually, Eject tool: (0) No<br>3 return value(s) . |
| 220 | Sequence: Set Current Position<br>current position of sequence 'ML_STAR._LidAgar' = '1' |
| 221 | _Sub05_TransportLid_Agar of KompetenteZellen_v1.1.0<br>_Sub05_TransportLid_Agar(ML_STAR._LidAgar, ML_STAR._Plate_Agar_StorePlated_Lids) |
| 222 | Grouping<br>Transfer Agarplate to Cytomat |
| 223 | iSWAP Transport on ML_STAR<br>Transport labware from 'ML_STAR._Plate_AgarTransport' to 'ML_STAR._move_Tecan'<br>1 return value(s) . |
| 224 | Position_Tecan of BVTlib_LTU<br>Position_Tecan() |
| 225 | HSL code.<br>sShellCmd = "C:\Dokumente und Einstellungen\Peter Neubauer\Eigene Dateien\Sebastian\Programms\Remote_Trick\ClientConsole.exe C:\Users\BVT-Administrator\Anaconda3\python.exe C:\Tecan_Scripts\Remoting\CytomatAgarplate.py -m 0 -s " + IStr(main_CytomatPos);<br>Trace(sShellCmd);<br>Shell(sShellCmd, 2,1); |
| 226 | Assignment with Calculation<br>'main_CytomatPos' = 'main_CytomatPos' + '1' |
| 227 | Grouping |
| 228 | End Loop |
| 229 | HSL code.<br>Trace("Plating done"); |
| 230 | Grouping |
| 231 | |

Figure S3: Tecan LHS

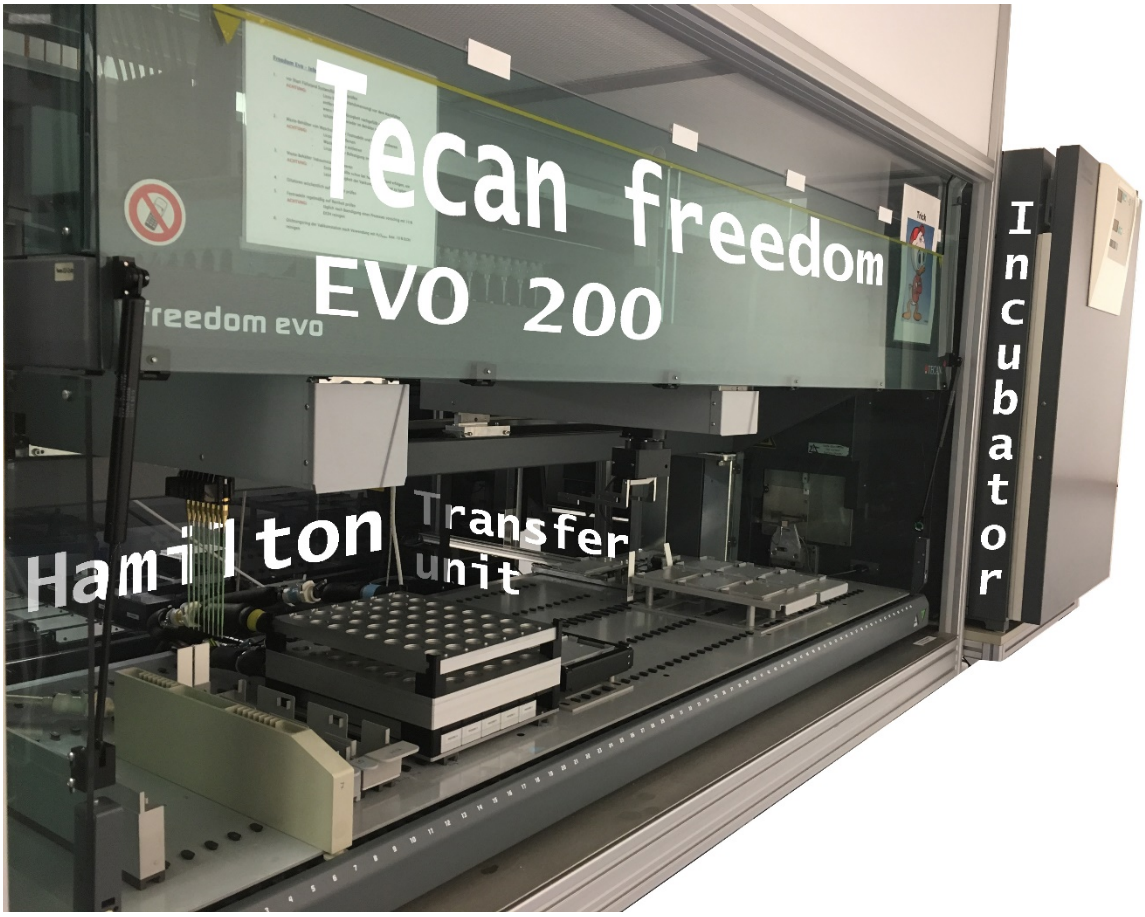


Figure 1: Second used Liquid handling station (LHS). Plates are stored at the incubator on the right site of the LHS. On a command of the Hamilton LHS a plate is moved from the incubator to the Transfer unit. A second command was used to return the plate from the transfer unit into the incubator.

## Source Code S4 MATLAB Source Code

```
function DiluteForChemostat(iRunID)
sim = 0;
%% Ziel OD:
    fTragetOD = 0.8;
    iCultivationVol = 170; % [µL]

%% Connect to ilab
    if ispc % check if running on Windows
        ilab = actxserver('BVT_iLabDriver.ilab_net_class');
% %         ilab = actxserver('ILAB_COM_BVT.ILAB_COM_BVT');
    else
        ilab = iLab_driver_universal;
    end
    ilab.SQL_Close;
    ilab.SQL_Connect;

%     ilab.run_id = iRunID;
%     ilab = ilab.get_bioID(iRunID);
%     iExpID = ilab.getExpIDByBioID();
%     iProfID = ilab.getProfIDByBioID();
    iBioID = ilab.getBioreactorIDs(iRunID);
    iBioID = iBioID(1);
    iExpID = ilab.getExperimentIDs(iBioID);
    iProfID = ilab.getProfilIDs(iBioID);
    iNumOfExperiments = length(iExpID);



    %% Calling Measurments
    fOD600 = ilab.getExperimentMeasurements(iExpID,'OD600');
    fTime = fOD600(:,1)/3600;
    fOD600 = fOD600(:,2:end);

    iSizeOfData = size(fOD600);
    iNumOfMeasurments = iSizeOfData(1);

    %% get last Setpoints to compute the last dilution factor
    fDilutionOld =   ones(1,length(iExpID));
    if iNumOfMeasurments > 1
        for ci = 1:iNumOfExperiments
            fDilutionOld(ci) = (iCultivationVol-ilab.SetpointgetCurrent(iProfID(ci),
'Puls_Medium'))/iCultivationVol;
        end
        % Berechne vorletzte OD neu:
        fOD600(end-1,:) = fOD600(end-1,:).*fDilutionOld;
    end

    %% create timestamp:
    iTime = ilab.getCultivationTime(iRunID);

    %% Compute µ / OD set
    % Erstelle Array's
    fODNext =       zeros(1,length(iExpID));
    fDilution =  ones(1,length(iExpID));
    fVolRemain =  zeros(1,length(iExpID));
    fVolAdd =       zeros(1,length(iExpID));
    fVolRemove =    zeros(1,length(iExpID));

    fMu = zeros(iNumOfMeasurments-1, length(iExpID));
    if ~(iNumOfMeasurments > 1) % if no µ values can be calculated
        for ci = 1:length(iExpID)
            fVolAdd(ci) = 20;
            fVolRemove(ci) = 0;
            if ~sim
                ilab.SetpointSet(int32(iProfID(ci)), 'Puls_Medium', iTime, fVolAdd(ci));
                ilab.SetpointSet(int32(iProfID(ci)), 'Probe_Volume', iTime,
fVolRemove(ci));
            end
        end
    else % normal process ...
        for ci = 2:iNumOfMeasurments
            for cj = 1:length(iExpID)
```

```
                fMu(ci, cj) = (log(fOD600(ci,cj))-log(fOD600(ci-1,cj))) / (fTime(ci)-
fTime(ci-1));
            end
        end
        for ci = 1:length(iExpID)
            fODNext(ci) = fTragetOD / exp(fMu(end,ci) * (fTime(end)-fTime(end-1)));
            if fODNext(ci)~=0
                fDilution(ci) = fODNext(ci) / fOD600(end, ci);
            end
            fVolRemain(ci) = fDilution(ci) * iCultivationVol;
            if fVolRemain(ci) > (iCultivationVol - 20)
                fVolRemain(ci) = (iCultivationVol - 20);
            elseif fVolRemain(ci) < 45
                 fVolRemain(ci) = 45;
            end
            % Compute Volumes
            fVolRemove(ci) = (iCultivationVol - 20) - fVolRemain(ci);
            fVolAdd(ci) = iCultivationVol - fVolRemain(ci);
            %% Write data to database
            if ~sim
                ilab.SetpointSet(int32(iProfID(ci)), 'Puls_Medium', iTime, fVolAdd(ci));
                ilab.SetpointSet(int32(iProfID(ci)), 'Probe_Volume', iTime,
fVolRemove(ci));
            end
        end
    end
end
```
